# Molecular Characterization of Infectious Bronchitis Virus Strain HH06 Isolated in a Poultry Farm in Northeastern China

**DOI:** 10.3389/fvets.2021.794228

**Published:** 2021-12-16

**Authors:** Ghulam Abbas, Yue Zhang, Xiaowei Sun, Huijie Chen, Yudong Ren, Xiurong Wang, Muhammad Zulfiqar Ahmad, Xiaodan Huang, Guangxing Li

**Affiliations:** ^1^Heilongjiang Key Laboratory for Animal and Comparative Medicine, College of Veterinary Medicine, Northeast Agricultural University, Harbin, China; ^2^College of Pharmaceutical Engineering, Jilin Agriculture Science and Technology University, Jilin, China; ^3^Department of Computer Science and Technology, College of Electrical and Information Technology, Northeast Agricultural University, Harbin, China; ^4^State Key Laboratory of Veterinary Biotechnology, Harbin Veterinary Research Institute, Chinese Academy of Agricultural Science, Harbin, China; ^5^Department of Plant Breeding and Genetics, Faculty of Agriculture, Gomal University, Dera Ismail Khan, Pakistan

**Keywords:** molecular epidemiology, zoonosis, coronaviruses, infectious bronchitis virus, evolution

## Abstract

Spike (S) glycoprotein is an important virulent factor for coronaviruses (CoVs), and variants of CoVs have been characterized based on S gene analysis. We present phylogenetic relationship of an isolated infectious bronchitis virus (IBV) strain with reference to the available genome and protein sequences based on network, multiple sequence, selection pressure, and evolutionary fingerprinting analysis in People's Republic of China. One hundred and elven strains of CoVs i.e., *Alphacoronaviruses* (Alpha-CoVs; *n* = 12), *Betacoronaviruses* (Beta-CoVs; *n* = 37), *Gammacoronaviruses* (Gamma-CoVs; *n* = 46), and *Deltacoronaviruses* (Delta-CoVs; *n* = 16) were selected for this purpose. Phylogenetically, SARS-CoV-2 and SARS-CoVs clustered together with Bat-CoVs and MERS-CoV of Beta-CoVs (C). The IBV HH06 of Avian-CoVs was closely related to Duck-CoV and partridge S14, LDT3 (teal and chicken host). Beluga whale-CoV (SW1) and Bottlenose dolphin-CoVs of mammalian origin branched distantly from other animal origin viruses, however, making group with Avian-CoVs altogether into Gamma-CoVs. The motif analysis indicated well-conserved domains on S protein, which were similar within the same phylogenetic class and but variable at different domains of different origins. Recombination network tree indicated SARS-CoV-2, SARS-CoV, and Bat-CoVs, although branched differently, shared common clades. The MERS-CoVs of camel and human origin spread branched into a different clade, however, was closely associated closely with SARS-CoV-2, SARS-CoV, and Bat-CoVs. Whereas, HCoV-OC43 has human origin and branched together with bovine CoVs with but significant distant from other CoVs like SARS CoV-2 and SARS-CoV of human origin. These findings explain that CoVs' constant genetic recombination and evolutionary process that might maintain them as a potential veterinary and human epidemic threat.

## Introduction

Coronaviruses (CoVs) are a group of RNA viruses that mainly infect respiratory systems of domestic and wild birds as well as mammals including humans. These viruses belong to the subfamily *Orthocoronavirinae* of the family *Coronaviridae* (1, 2), further classified into *Alphacoronavirus, Betacoronavirus, Gammacoronavirus*, and *Deltacoronavirus* genera (3). The CoVs are enveloped viruses with a helical-symmetry nucleocapsid that projects club-shaped spikes. The genome is a positive-sense single-stranded RNA of 26-32 kilobase pairs that encodes main structural proteins i.e., spike (S) glycoprotein comprising 2 subunits (S1 and S2), envelop (E) protein, membrane (M) protein, and nucleocapsid (N) protein (4–6). The S glycoprotein is an important virulent factor i.e., plays role in viral adsorption and invasion into the host cells (7, 8). The evolution of S protein is more active and it often undergoes mutation. The changes of certain amino acids influence the conformation of antigenic determinants, resulting in the generation of new strains (9). Usually, difference of amino acids in S1 by 20~50% is considered for different serotypes, however in some instances, only 2% or 10~15 amino acids variation may lead to the emerging of different serotypes of infectious bronchitis virus (IBV) (10, 11). Hence, evolution process is considered important factor which plays major role in many emerging serotypes. Indicating the positions of amino acids evolutionary conservation is important for maintaining the protein structure and function (12, 13). Therefore, detection of selected sites may enlighten the selection forces and detects the functionally significant sites for CoVs S protein interaction.

IBV was the first coronavirus described, and was found by Schalk and Hawn (1931) in North Dakota of the United States of America (USA) (14). After that, the related CoVs have been isolated from other birds, mammals, and rodents (15); however, the first CoV in a human was identified in the 1960s and was associated with the common cold (16, 17). In the last couple of decades, disease pandemic viruses Severe Acute Respiratory Syndrome Coronavirus (SARS-CoV) and Middle East Respiratory Syndrome (MERS) have caused a larger number of mortalities (18, 19). The last few days of the year 2019 bared the advent of a pathogenic disease caused by a novel epidemic of CoV (Severe Acute Respiratory Syndrome 2; SARS-CoV-2) (20). During the manifestation of disease, the CoVs has broader tissue tropism, mainly toward respiratory system, however can potentially infect other organ systems e.g., gastrointestinal and reproductive tract (21, 22). *Avian coronavirus* (Avian-CoV) from the genus *Gammacoronavirus* causes avian infectious bronchitis which is highly infectious, and affects respiratory, renal, and reproductive system. It causes significant drop in weight gain (in broilers) and egg production (in layers) (10, 23). Though, chickens (*Gallus gallus*) are considered natural hosts of IBV, these viruses have been reported to cause enteric diseases in turkeys (Turkey-CoV) (24), renal and respiratory disease in pheasants (Pheasant-CoV) (25). Duck coronavirus (Duck-CoV) (26), peafowl coronavirus (PeF-CoV) (27), pigeon coronavirus (Pi-CoV) (28), Canada goose coronavirus (GCoV) (29) seem to be less pathogenic. However, the host range might be even broader e.g., swans, mallards, geese, and gulls also exhibited IBV-like symptoms and yielded viruses that had gene fragments from M41, 793B, and QX lineages (25).

During replication, Avian-CoVs have high genetically recombination potential (30). Genetic techniques have played an important role in understanding the genetic relatedness of different microorganisms, pathogens, and the diseases caused by the pathogens as well as their evolutionary mechanism (31, 32). Recent pandemic of COVID-19 has drawn attention to the potential zoonotic threats of the CoVs (33, 34). In order to control and prevent the occurrence of such pandemics, it is important to understand the virus origin, genetic mechanics and its mode of transmission between intra-host species. In our study, we presented phylogenetic relationship of an isolated IBV strain with reference to the available genome and protein sequences based on phylogenetic, recombinant network, multiple sequence, selection pressure, and evolutionary fingerprinting analysis.

## Materials and Methods

### Chicken Embryos

Specific-pathogen-free (SPF) chicken embryos were used for virus isolation and titration. 9–11-day-old SPF chicken embryos were purchased from the Experimental Animal Center of the Harbin Veterinary Research Institute (HVRI), Chinese Academy of Agricultural Sciences, People's Republic of China (PRC).

### IBV HH06 Isolation and Identification

The IBV isolate HH06 (GenBank accession number MH181793.1) was isolated from Hy-Line chicken suspected of having infectious bronchitis infection in a farm at Northeastern China and kept in the Veterinary Pathology Laboratory of the College of Veterinary Medicine, Northeast Agricultural University as earlier described by Ren et al. (35). Briefly, the purification and propagation of the isolate was done by three times passaging in allantoic cavity of 9-day-old SPF embryonated chicken eggs (ECE) and distinct IBV characteristics e.g., embryo dwarfing, hemorrhages, curling or stunting of embryos were observed (36). The 50% embryo infectious dose (EID_50_) was measured by inoculating 10-fold dilutions in groups of 9-day-old ECE as described previously (37).

### Viral RNA Extraction and Reverse Transcription Polymerase Chain Reaction

RNA was extracted from the allantoic fluid using TRIzol reagent (TaKaRa, Dalian, China), according to the protocol of manufacturer. Reverse transcriptase reaction was performed according to procedures provided by Qiagen RT-PCR kit. Briefly, a total of 20 μl mix was prepared as follows; 8 μL DEPC, 4 μl 5×RT-buffer, 1 μl dNTP, 1 μl Oligo (dT), 5 μl RNA, 0.5 μl m-MLV, and 0.5 μl RNase. After preparation of cDNA, IBV-N primers (189 bp) [Sense: CAAGCTAGGTTTAAGCCAGGT; Antisense: TCTGAAAACCGTAGCGGATAT] (38) were used for RT-PCR IBV detection. PCR reactions included initial denaturation for 95°C for 5min, followed by 40 cycles of denaturation for 30 sec at 94°C, annealing for 30 sec at 55.7°C, and extension for 2 min, at 72°C and a final extension cycle at 72°C for 10 min with holding temperature of 4°C. PCR products were run on 1% agarose gel electrophoresis for confirmation and visualized by subsequent UV trans-illumination (Bio-Best 140E, SIM, USA) (39).

### Cloning and Sequencing of Target Gene

Gel Extraction Mini Kit (Omega, USA) was used for DNA purification and recovery of the PCR products. Purified PCR products ligated with a TA cloning vector pMD18-T (TaKaRa, Japan) were transformed into competent *E. coli* cells strain JM109 (Beijing TransGen Biotech, PRC). Confirmation of clones containing recombinant plasmid was achieved by PCR and restriction enzyme digestion. The PCR conditions were the same as that for the above-mentioned PCR amplification. Three positive clones were randomly selected and cultured. Recombinant plasmids were sequenced at Shanghai Sang-gong Biological Engineering Technology & Services Co., Ltd (Shanghai, China).

### Genetic, Phylogenetic, Motif Analysis, and Comparative Sequence Alignment

A total of 111 corona viruses from the *Coronaviridae* family were selected to analyze phylogeny and genetic relatedness. The sequence of IBV strain HH06 (GenBank number MH181793.1) isolated in this study along with 110 viruses were aligned using ClustalW multiple alignment algorithm. The phylogenetic tree was constructed based on a maximum-likelihood method (JTT model) using the MEGA 7.0 version with bootstrap replicates (1,000) (https://www.megasoftware.net) (40). The sequences from GenBank (https://www.ncbi.nlm.nih.gov/genbank), which represent the well-established four genera of *Coronaviridae* are enlisted in Supplementary Table S1. The motif analysis was performed using the protein sequence of the S genes through the online database the MEME (https://meme-suite.org/meme/tools/meme). The ClustalW in MEGA 7.0 was used to align the amino acid sequence of S proteins of 31 CoVs representing different hosts, origins, and genotypes among all selected 111 CoVs of four genera of the *Coronaviridae* family (Supplementary Table S1). Aforementioned sequences were subjected to the GeneDoc program to shade the conserved amino acids in alignment (40).

### Recombinant Network Tree

The spike protein sequence was analyzed to evaluate the degree of possible recombination. A network tree was assembled from protein sequences alignment of IBV strain HH06 and 110 reference CoV strains from different genera by using the SplitTree 4.13.1 (http://www.bio-soft.net/tree/SplitsTree.htm) (41).

### Selection Pressure Analysis

Online database SELECTION (https://selecton.tau.ac.il/) was used to ratify codon sites under selection pressure. Aligned codon sequence of CoVs proteins was tested in the SELECTION that allows shifting the ω ratio between different codons within the aligned sequence and this was measured by maximum-likelihood test through Bayesian inference method (42). Moreover, the selection results are shown with color scales demonstrating various types of selection. The identification and accession numbers of protein coding sequence of gene (CDS) are presented in Supplementary Table S1.

### Evolutionary Fingerprinting

We used the EFP model to represent evolutionary fingerprints as probability distributions and presented a methodology for comparing these distributions in a way that is robust against variations in data set size and divergence. The EFP was done by using an online Data Monkey classical tool (https://www.datamonkey.org/) (43) on the aligned CDS sequences of selected CoVs including SARS-CoV-2, SARS-CoV, MERS –CoV, and IBV.

### Evolutionary Analysis of Diversifying Selection

Neutrality analysis was done based on maximum likelihood computation of dN-dS using the HyPhy software program implemented in MEGA 7.0, using the Nei-Gojobori method (44). All position gaps and missing data were eliminated. The evolutionary history was inferred using the maximum likelihood method based on the Kimura 2-parameter model and the phylogenetic tree of CoVs S gene was constructed using MEGA 7.0 software package based on maximum likelihood (45). ClustalW software was used for genetic sequence and the similarity analysis of S genes. For each codon, estimates of the numbers of inferred synonymous (s) and non-synonymous (n) substitutions are presented along with the numbers of sites that are estimated to be synonymous (S) and non-synonymous (N). These estimates were produced using the joint Maximum Likelihood reconstructions of ancestral states under a Muse-Gaut model of codon substitution and Tamura-Nei model of nucleotide substitution (43). For estimating ML values, a tree topology was automatically computed. The test statistic dN–dS is used for detecting codons that have undergone positive selection, where dS is the number of synonymous substitutions per site (s/S) and dN is the number of non-synonymous substitutions per site (n/N). A positive value for the test statistic indicates an overabundance of non-synonymous substitutions. In this case, the probability of rejecting the null hypothesis of neutral evolution (*p*-value) was calculated (46). Values of *p* < 0.05 are considered significant at a 5% level and are highlighted. Normalized dN–dS for the test statistic is obtained using the total number of substitutions in the tree (measured in expected substitutions per site). It is useful for making comparisons across data sets. Maximum Likelihood computations of dN and dS were conducted using HyPhy software package. The analysis involved 110 nucleotide sequences. Codon positions included were 1st+2nd+3rd+Noncoding. All positions containing gaps and missing data were eliminated. There were a total of 2,625 positions in the final dataset. Evolutionary analyses were conducted in MEGA7 (40). Evolutionary analysis of diversifying selection was performed by various approaches to detect the episodic diversifying detection affecting individual codon sites. Mixed-effects model evolution (MEME) combines the fixed effects to identify instances of both episodic diversifying selection and pervasive positive selection at the individual branch site level using Markov Chain Monte Carlo (MCMC) routine, which ensures the robustness against model misspecification over predefined sites through approximate Bayesian method (47). The fitting of MEME to alignment, MG94xREV codon model, was applied using parameter estimates ω = β/α fitted to the data using the GTR nucleotide model as initial values. The selective pressure was measured with two parameters β: β- < α and β+ and the alternative model include four parameters for each site: β-, β+, and α estimating site to site substitution variability rates 42]. The values of *p* < 0.05 were considered as significant from the LRT based on χ2 asymptotic distribution (44).

## Results

### Isolation, Identification, and Confirmation of IBV HH06

IBV strain HH06 was isolated using 9–11-day-old SPF ECE. The morphology and gross changes were observed, dwarfism, hemorrhage, and congestion were found (Supplementary Figure S1). The allantoic fluid was harvested. The presence of IBV HH06 was confirmed by RT-PCR using IBV-N specific product (189 bp) primers (Supplementary Figure S2).

### Genetic, Phylogenetic, and MOTIF Analysis of the S Protein

**Figure 1:**
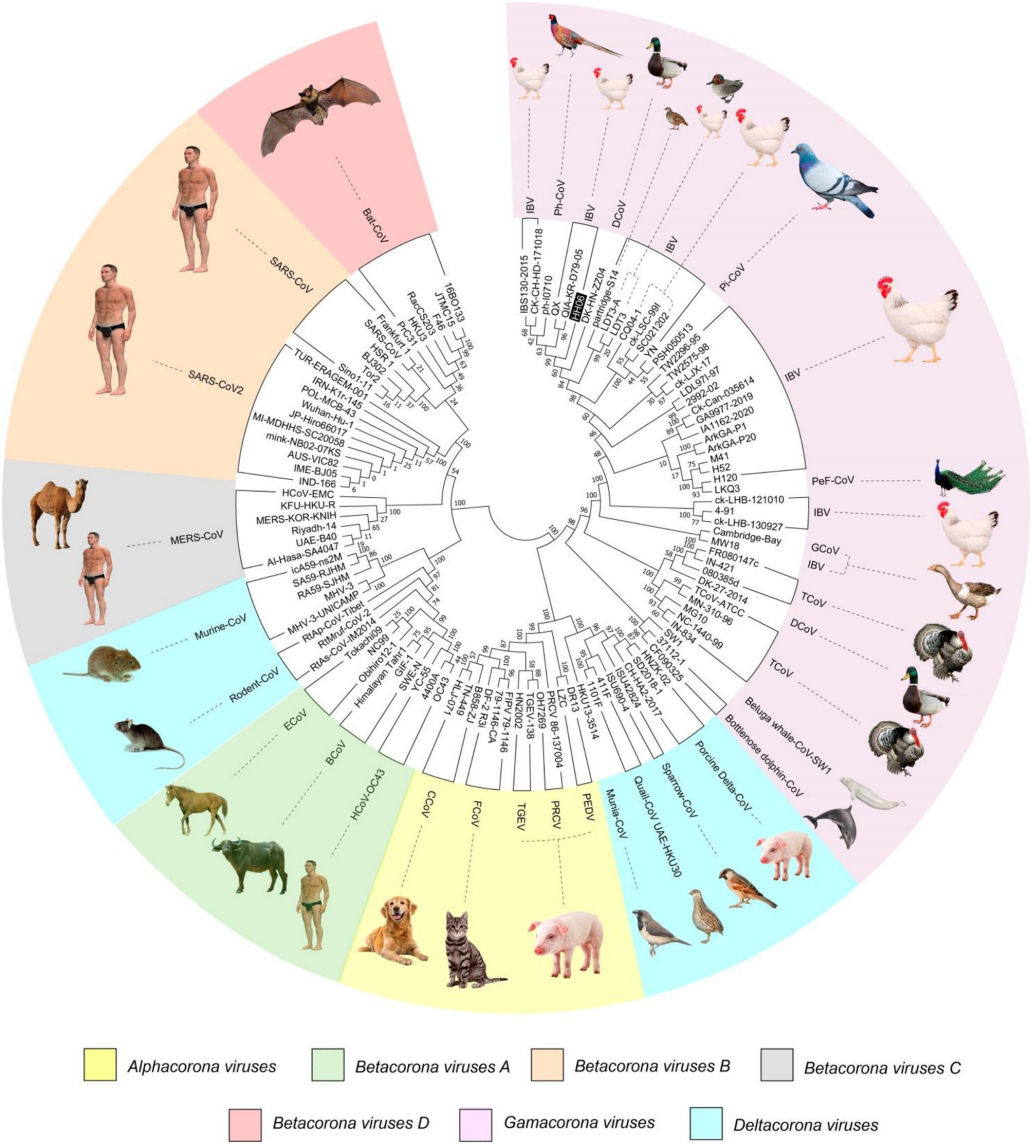
A phylogenetic tree was constructed based on the Spike (S) gene from the four genera (Alpha, Beta, Gamma, and Delta coronaviruses) of *Coronaviridae* family comprising IBV HH06 and 110 reference CoV strains using the Maximum-Likelihood Method of MEGA7.0 version with bootstrap replicates of 1,000. The selected reference strains and their natural host are represented with a different color.

**Figure 2:**
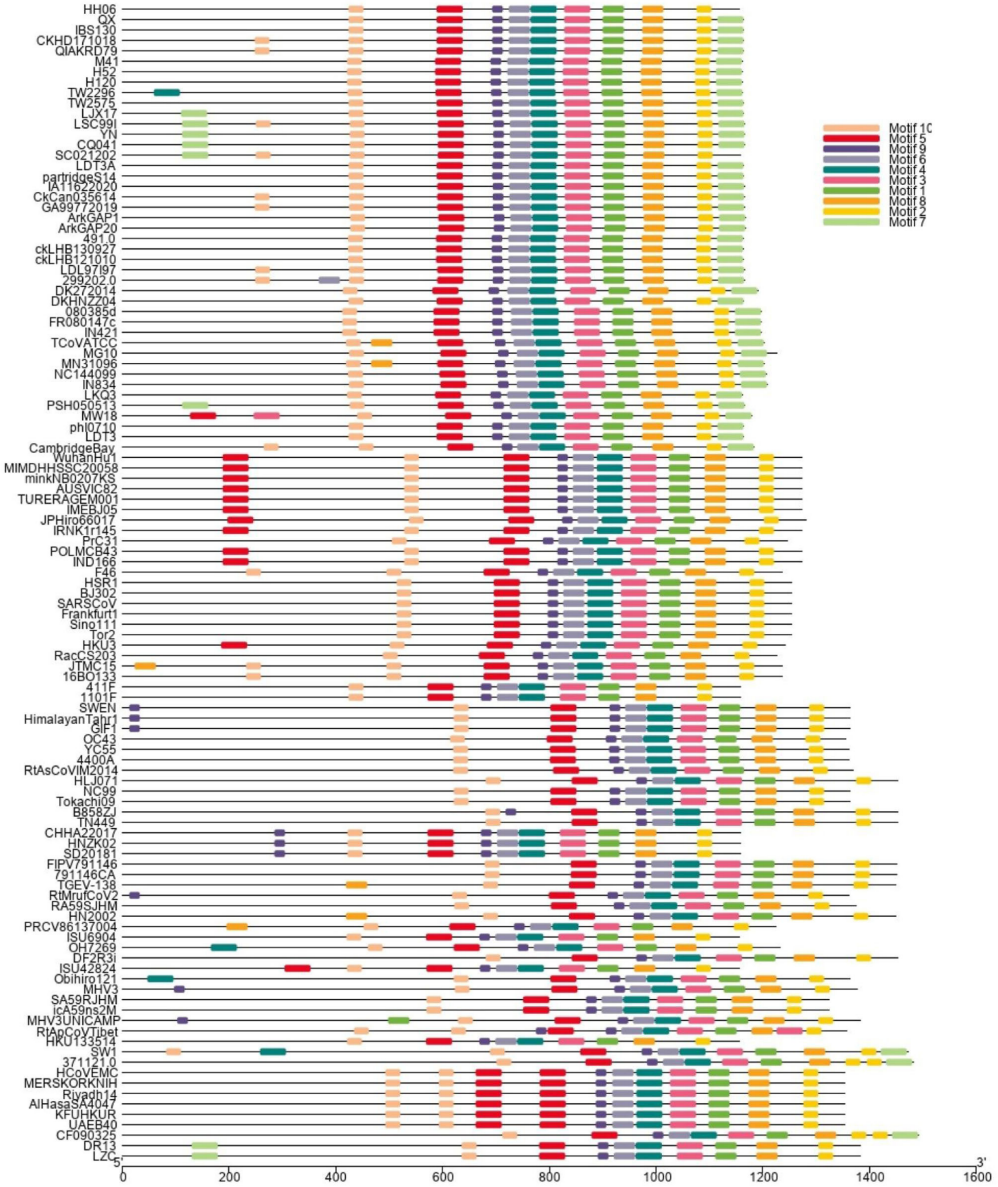
Different colors display the motifs of Spike (S1 and S2) protein. The spike proteins are listed according to genera and their phylogenetic relationships.

A phylogenetic tree (Figure 1) was constructed based on amino acid CDS of S glycoprotein to assess the genetic relevancy and discrimination among the four main genera (*Alphacoronavirus, Betacoronavirus, Gammacoronavirus*, and *Deltacoronaviruse* of the *Coronaviridae* family) comprising different mammalian and avian coronaviruses. Among selected viruses were SARS-CoV-2, SARS-CoV, and MERS-CoV of mammalian origin along with IBV strain HH06 of avian origin isolated in this study. The IBV strain HH06 clustered into the GI-19 genotype (QX-type) of Avian-CoVs that belongs to *Gammacoronavirus* and was closely related to Duck-CoV DK/CH/HN/ZZ2004 (GenBank accession number AEO86768.1) and partridge S14 (GenBank accession number AAT70772.1), LDT3 (GenBank accession number AAU14248.1) (teal and chicken host) of GI-18 (LDT3-A). Ph-CoV strains ph/China/I0710 (GenBank accession number QDA76255.1) and PSH050513 (GenBank accession number AAZ85066.1) of Avian-CoVs also clustered closely in the same group. These indicate the intra-host evolution of Avian-CoVs from one genotype to another and from one host to another host. The S gene glycoprotein sequence of Beluga whale-CoV SW1 (GenBank accession number ABW87820.1) and Bottlenose dolphin-CoVs 37112-1 (GenBank accession numbers QII89019.1) and HKU22 (GenBank accession numbers 211 AHB63481.1) clustered at distant; however, making group along with Avian-CoVs altogether into *Gammacoronaviruses*. Another sequences set of delta-CoVs that comprises sparrow CoV, munia CoV, and Quail-CoV of Avian-CoVs along with porcine delta-CoV clustered together with Feline CoVs, Canine CoVs. PRCV, TGEV, and PEDV belonging to alpha CoVs, hence making a discrete cluster covering the coronaviruses from avian and mammalian species. A cluster of exactingly CoVs, branch off into the human CoVs (HCV-OC43), and a cluster containing murine CoVs, equine CoVs, and rodent CoVs. The MERS viruses of Beta-CoVs (C) were closely associated with SARS-CoVs-2 and SARS-CoVs. In the same manner, Bat-CoVs show close association with SARS-CoVs. Currently, the S gene mainly determines the serotype and tissue tropism of different virus strains. The conserved domains of spike proteins are determined by the MEME (Figure 2). In total, we found ten motifs in the S gene, and their annotations confirmed through the Pfam databases. All S protein motifs are generally well-conserved and similar within the same phylogenetic class; however, variation was also observed (Supplementary Table S2).

### Recombinant Network Analysis

**Figure 3:**
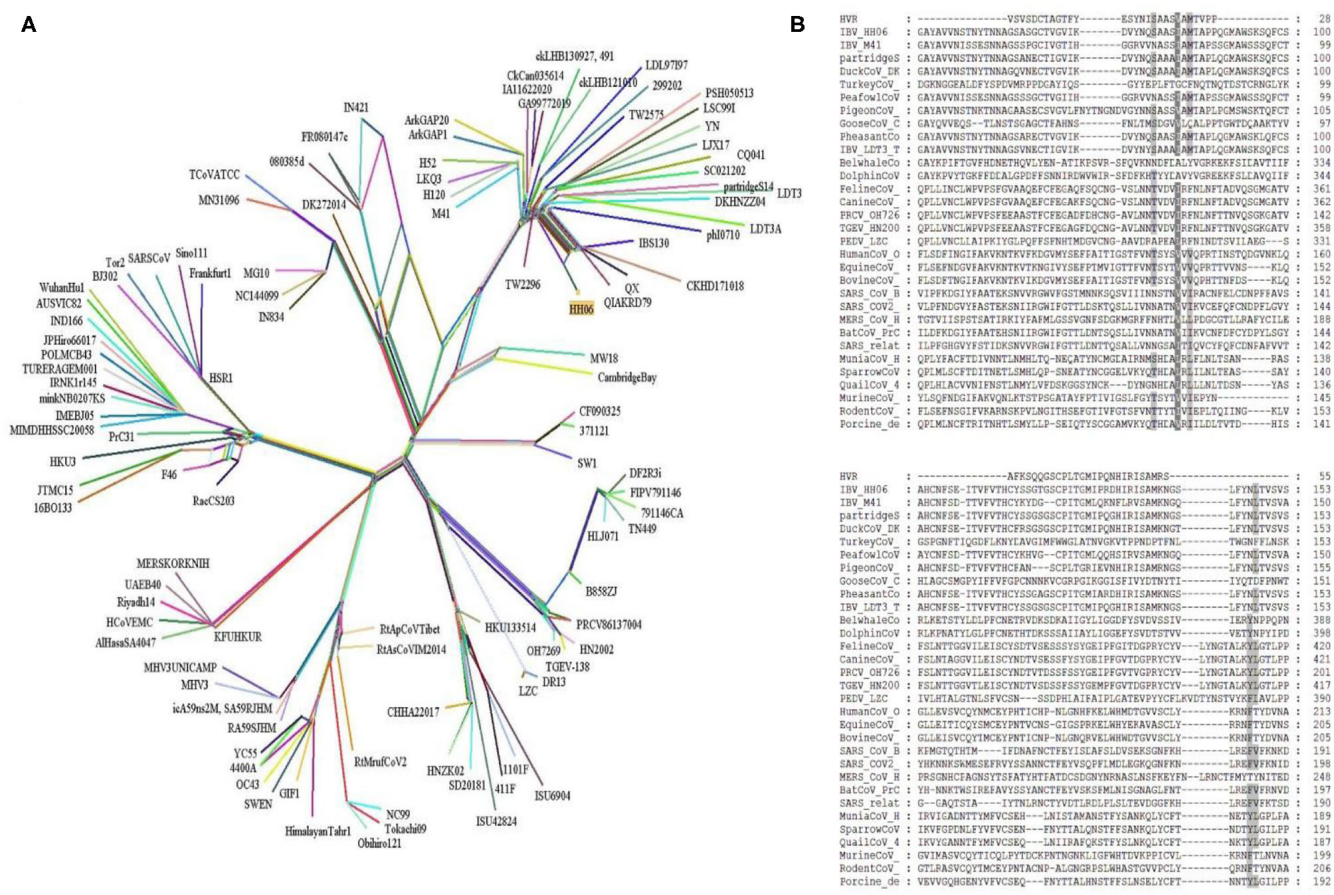
A Network tree among 111 coronaviruses (see Supplementary Table S1) was constructed from protein sequence alignments of spike protein using the SplitsTree 4.14.5. **(A)** The multiple reticulate networks indicate the recombination events among different coronaviruses. The isolate (HH06) of this study is highlighted in yellow squire. The other main reference sequences of SARS-CoV2, SARS-CoV, and MERS-CoVs were also included. **(B)** Sequence alignment of S proteins from four genera of coronaviruses. Amino acid sequence alignment of 32 S proteins was done with the MEGA7. Further, the MEGA7 alignment was used in the GeneDoc program to shade the identical and similar amino acids among all alignments. Dark shad represents identical amino acids and the gray shade indicates similar amino acids.

Further, a recombinant network tree was generated (Figure 3A) of 111 selected strains from four genera (Alpha, Beta, Gamma, and Delta) of CoVs comprising human, animals, and avian origin. The CoVs of human origin distributed in separate clades. The *Betacoronaviruses* B (SARS-CoV-2 and SARS-CoV) and *Betacoronaviruses* D (Bat-CoVs) shared common clades, although branched differently, however possible to be originated from bats. The MERS-CoVs of camel and human origin from *Betacoronaviruses* C distributed in individual clade, however, was closely associated with *Betacoronaviruses* B and D. Although human coronavirus (HCoV-OC43) has a human origin, it branched together with Bovine-CoVs and showed a significant distance from other human CoVs like SARS-CoV-2 and SARS-CoV. Feline- and Canine-CoVs showed common origin thus branched together, however, were significantly distant from other SARS-related viruses. The *Gammacoronaviruses* of avian origin indicated association at a distant place from *Betacoronaviruses* (SARS-CoV2, SARS-CoV, Bat-CoV and MERS-CoVs), however, Beluga whale-CoV strain SW1 and Bottlenose dolphin-CoVs strains 37112-1 and CF090325 of animal origin differentiated into separate clade from other CoVs of animal origin and clustered at distant however making group along with Avian-CoVs altogether into *Gammacoronaviruses*. Isolate HH06 from the current study was clustered into Avian-CoVs group, mainly of chicken origin, however, Duck-CoV and Pheasant-CoV showed a close relationship with the HH06 isolate from chicken. Similarly, Turkey-CoVs separated clade from other avian mainly chicken however 3 Turkey-CoVs differentiated into a separate clade.

### Comparative Sequence Alignment

Alignment of interrelated amino acids sequences was performed in comparison to 4 genera of coronaviruses and these isolates expressed distinctive amino acids mutations in the HVRs compared to different species. The result of the alignment of the S gene of IBV isolate HH06 from this study shown the occurrence of numerous mutational sites (Figure 3B). The majority of mutation sites were located in HVRI in spike protein structure that was comparable to sequence alignment of amino acids of selected 31 CoVs of 4 genera of *Coronaviridae* family.

### Selection Pressure Analysis by the Position of Amino Acids

**Figure 4:**
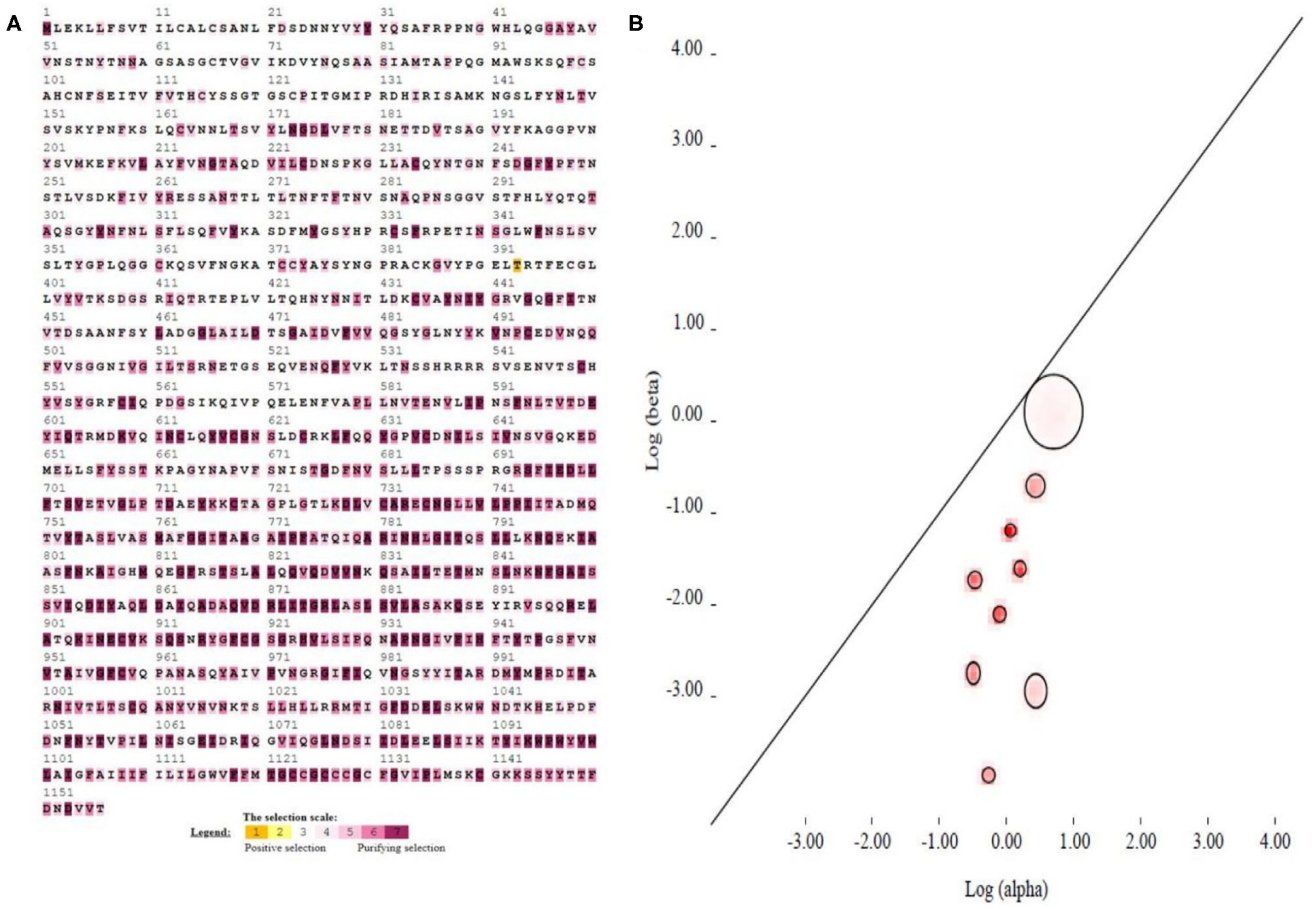
Evolutionary mechanism of 111 coronaviruses. **(A)** The implementation of the MEC Model, which is found in the Selection Server, is used for selection pressure analysis of the viral spike protein sequences. The codons, which are highlighted with brown and yellow colors, represent positive selection, while white and gray colors are for the neutral codons. On the other hand, the codons, which are highlighted with purple, represent the purifying selection. **(B)** Evolutionary fingerprint (based on 1,000 distribution samples). The plot depicts the estimate of the distribution of synonymous and non-synonymous rates inferred from this alignment (on the log-log scale). The ellipses reflect a Gaussian-approximated variance in each individual rate estimate, and colored pixels show the density of the posterior sample of the distribution for a given rate. The diagonal line represents the idealized neutral evolution regime (*ω* = 1), points above the line correspond to positive selection (ω > 1), and points below the line–to negative selection (ω < 1).

The selection pressure test was performed in the SELECTION server (http://selecton.tau.ac.il/) that uses the Mechanistic Empirical Combination (MEC) model for estimating the selection pressure at particular codons. The MEC model takes into account the variances between amino acid substitution rates. Adaptive selection pressure was found at various codons in S protein (Figure 4A), identified under positive selection. A total of 36.63% amino acids showed positive selection while the rest of the amino acids were under purifying process (Figure 4A). The result suggested that all gene pairs have evolved mainly under the influence of purifying selection.

### Evolutionary Fingerprinting

Evolutionary fingerprinting of spike gene was inferred through codon model evolution based on synonymous and non-synonymous substitution rates using genetic algorithm. The probability of site-to-site distribution of synonymous and non-synonymous substitution rates exploited the ratio ω = β/α, which is estimated on the basis of the likelihood log and Akaike information criterion (AIC) using five rate classes identified neutral evolution at spike gene. The likelihood log was−129131.88712 for the S gene by using nine class rates with 49 parameters, and the AIC value was 258361.77424. The non-synonymous to synonymous ratio *ω* = β/α values for all nine classes of CoVs were 0.004, 0.022, 0.117, 0.275, 0.077, 0.151, 0.262, 0.312, and 0.543, respectively (Supplementary Table S3). The graph showing the neutral evolution and only a few sites under the circle above diagonal has positive evolution at the S gene (Figure 4B).

### Positive and Purifying Selection

There were 2,625 coding positions in the nucleotide sequences of the S gene and the coding positions were included 1st, 2nd, 3rd, and non-coding. Among 2,625 positions only 18 positions from codon 26 to 866 had undergone positive selection, as the values dN-dS > 1 indicated positive selection. The number of synonymous and non-synonymous mutations along with synonymous and non-synonymous substitution sites was also given. The values of dN-dS were estimated for natural selection. The number of synonymous and non-synonymous codon changes, as well as the number of potential synonymous and non-synonymous changes for all sequences in pairwise comparison, was counted (Supplementary Table S4).

The MEME analysis detected 46 sites undergone episodic diversifying selection. All the 46 sites were detected under episodic diversifying having *p-*values <0.01 (Table 1). This model also estimates the synonymous (α) and non-synonymous (β) substitution rates, and the sites having values β > α were considered as significant and determined these sites under diversifying selection. The sites inferred to have experienced pervasive non-synonymous substitution throughout the evolutionary history with *p*-values <0.01, and this site evolved with β+ > α, as it is under positive selection in all analyses providing strong evidence of positive selection of S gene, whereas all other sites were conserved. The value of q was determined using the Simes' method to reduce the false discovery rate under a strict neutral null model (Table 1).

**Table 1:** Mixed-effect model evolution (MEME) based episodic diversifying selection of S genes.

**Codon**	**α**	**β^−^**	**Pr[β = β^−^]**	**β^+^**	**Pr[β = β^+^]**	***p-*** **value**	***q-*** **value**
23	0.763537	0.0876926	0.791942	6.94636	0.208058	0.00599763	0.306802
32	0.0303846	0.0303846	0.845357	16.0443	0.154643	0.000109085	0.0217625
183	0	0	0.808421	1385.75	0.191579	4.86022e-05	0.0121202
292	2.25206	0.196196	0.983314	1017.51	0.0166862	0.000440945	0.0488714
298	1.42265	0.174859	0.939828	100.502	0.0601715	7.95141e-06	0.0158631
317	1.04444	0.137501	0.887011	18.7376	0.112989	0.00021124	0.0301017
325	1.13163	0.232676	0.92076	20.307	0.07924	0.00463552	0.249942
342	1.57608	0.159258	0.95851	79.7256	0.04149	0.00362301	0.24093
353	1.29453	0.352534	0.924912	28.1257	0.0750881	0.0069351	0.345888
386	0.753244	0.0399839	0.800754	8.28076	0.199246	0.00114942	0.0997
425	0.410353	0.02881	0.823356	11.616	0.176644	0.00245846	0.169125
433	0.593223	0.231261	0.93788	78.4249	0.06212	0.000153183	0.0254667
439	0.479128	0.113517	0.964491	72.4365	0.0355088	0.00124469	0.103465
552	0.99667	0.123464	0.905014	19.789	0.0949856	0.00458789	0.254245
665	0.438912	0.068045	0.916963	16.7519	0.0830372	0.000346139	0.0431592
800	0.697221	0.0904626	0.896715	28.2982	0.103285	5.45339e-05	0.0120883
801	1.19665	0.104413	0.94502	59.5819	0.0549798	3.53549e-05	0.0141066
812	0.435179	0.0407753	0.982768	209.337	0.0172323	2.63072e-05	0.0174943
945	0.311495	0.311495	0.931781	1385.76	0.0682191	0.00160388	0.114277
1010	0.666632	0.0706994	0.794554	6.50562	0.205446	4.24713e-05	0.0121043
1036	1.07674	0.149688	0.807791	11.1189	0.192209	0.00872274	0.395497
1063	1.34172	0.051046	0.772181	29.3489	0.227819	0.000724438	0.0722627
1065	0.23116	0.0690992	0.628878	4.16857	0.371122	0.00766241	0.372842
1070	1.27829	0.088108	0.950218	156.206	0.0497822	0.00159718	0.118014
1089	0.461102	0.0974815	0.955559	39.1503	0.0444409	3.42666e-05	0.0170905
1171	0.702561	0	0.919435	13.1505	0.0805655	0.00835174	0.387482
1176	1.23305	0.10632	0.992475	1292.18	0.00752452	0.000136251	0.0247109
1241	0.43565	0.142802	0.823178	7.65695	0.176822	0.00110546	0.100245
1282	2.62966	0.0713705	0.935338	41.2155	0.0646616	0.000524567	0.0550796
1285	0.384711	0.103926	0.889866	15.0998	0.110134	3.81763e-05	0.0126936
1381	0.443969	0.102546	0.956052	54.664	0.0439479	2.62908e-05	0.0262251
1446	1.01357	0.0717032	0.938069	22.6903	0.0619309	0.00362951	0.233577
1519	0.586696	0.0355755	0.970597	186.959	0.0294032	0.000404232	0.0474378
1563	0.141133	0.0203677	0.958524	192.671	0.0414764	0.000164024	0.0251713
1606	0.906054	0.0786118	0.902833	9.24085	0.0971675	0.00768589	0.36508
1658	1.94819	0.170368	0.985206	210.228	0.0147941	0.00967217	0.419478
1694	0.331159	0.0278589	0.986102	432.483	0.0138985	0.00125178	0.0998924
1727	0.453456	0.155761	0.932596	22.5383	0.0674039	0.00387794	0.241765
1819	0.480708	0.031874	0.987011	49.0222	0.0129895	0.00435959	0.255805
1840	0.515394	0.0643477	0.980805	250.885	0.0191948	0.000251536	0.0334543
1866	0.776281	0	0.887619	12.702	0.112381	0.0055095	0.289249
1939	0.232632	0	0.978126	33.5588	0.0218743	0.00875951	0.388338
1942	0.500544	0	0.962909	25.6181	0.0370913	0.00152886	0.117311
1972	0.115119	0.0412817	0.894805	4.66122	0.105195	0.00396383	0.239631
1973	0.310806	0.0140975	0.96477	21.6742	0.03523	0.000802131	0.0762024
1989	0	0	0.846684	1.51907	0.153316	0.00440476	0.251072

## Discussion

Genetic techniques have played an important role in understanding the genetic relatedness of different microorganisms, pathogens, and the diseases caused by the pathogens as well as their evolutionary relatedness. In our study, we presented phylogenetic relationship of an isolated IBV strain HH06 with reference to the available genome and protein sequences based on network, multiple sequence, selection pressure, and evolutionary fingerprinting analysis. Recent pandemic of COVID-19 has drawn attention to the potential zoonotic threats of the coronaviruses.

Genetic-recombinant network-analysis helps understanding complex genetic relationships and evolution including viruses (48). Network analysis showed clustering of IBV HH06 strain with Duck-CoV and Pheasant-CoV which indicated an evolutionary relationship between these two viruses. Previously, same results were reported for Pheasant-Cov which indicate the susceptibility of IBV to these both host species (25, 49, 50). To infer sequence homology, alignment of interrelated amino acids sequences was done which indicated the majority of mutation sites in HVRI of the spike protein structure. Currently, CoVs are classified based on sequence comparison of structural protein, mainly spike protein gene (51). Spike protein facilitates the entry of virus to receptors present on the surface of host cells (52). These proteins have two cleaved forms called S1 and S2. The S1 plays a crucial role in the viral adsorption to the cellular glycoprotein receptor during the process of virus invasion into the host cell. The S2 play important role in fusion and forms stalk appearance to spike molecule. There is a very high conserved region in the C-terminal S2 mutation rate of S2 as it contains an extra cleavage site known as the furin S2' site, which directly influences the invasion mechanism of coronaviruses by host cells (53). The S protein is considered main determining factor in virus adaptation and tropism in the organs of host. Mutations that occurred in the S protein may influence the targeted host organ (54). We found 10 motifs in the S gene, and their annotations confirmed through the Pfam databases and assume their function in attachment and invasion/infection of the virus to the host cells.

A total of 36.63% positive selection on S protein amino acids indicated an influence of negative selection on all gene pairs. The selective removal of deleterious alleles that arise through random mutations can result in stabilizing selection. We selected Gaussian-approximate variance to evaluate the evolutionary fitness of our IBV strains based on S gene. It showed a majority of neutral evolution as compared to positive evolution on the S gene. To understand the dynamics of molecular sequence evolution, we estimated synonymous (α) and non-synonymous (β) substitution rates by MEME analysis, which indicated 46 episodic diversifying selection sites. In the present study, the phylogenic analysis indicated a broad mechanism, in which the MERS-CoVs of Beta-CoVs (C) were closely associated with SARS-CoVs-2 and SARS-CoVs of Beta-CoVs (B). In the same manner, Bat-CoVs show a close association with SARS-CoVs. Thus, similarly, in SARS-CoV-2 zoonotic transmission to humans, the viruses are considered to have originated in either bats or pangolins (31). Hence, the adaptation and recombination of SARS-CoV-2 have happened in another intermediate or reservoir host with the possibility of contact with pangolins or bats. Mutation and adaptation have determined the co-evolution of CoVs and their hosts (9). Gene recombination and mutation are both important means in producing multiple strains of CoVs, with multiple research reports on CoVs-recombination (55). There is a possibility of recombination among various strains, the recombination area and the antigenic profile of recombinant virus had important guiding significance for predicting the evolution and prevention of CoVs infection afterward (56, 57). Evolutionary conservation is critical for detecting amino acid positions and for sustaining the structural protein role. Consequently, during the selection pressure analysis, the sites that were detected as purifying and positive selection may instruct and clarify the spike protein gene function and evolution.

In the present analysis, purifying selection was observed during the selection pressure analysis. Li et al. (58) have reported purifying selection in different host species, along with frequent recombination among coronaviruses, proposes a common evolutionary mechanism that could lead to new emerging human coronaviruses. Similarly in our recombinant network tree analysis, the *Betacoronaviruses* B (SARS-CoV2 and SARS-CoV) and *Betacoronaviruses* D (Bat-CoVs) shared common clades, although branched differently, however possible to be originated from bats. Incidence of such intra-species transmission happenings in birds and mammals might be manifested by the predominant occurrence of CoVs at a large scale (59). In terms of Gamma-CoVs, thought provoking trends were observed during phylogenetic analysis. The Beluga whale- and Bottlenose dolphin-CoVs of mammals were closely associated with the branches of Avian-CoVs. Similarly, three avian-CoVs (sparrow, munia, and qual) were branched together with Porcine-CoV and this was suggestive of transmission among different species. Moreover, outcomes of the current analysis advocate that variation at the genomic and molecular level of the S gene sequence made viable CoVs adaptation to different host species. Additionally, the analysis of isolate from the present study IBV HH06 indicated that it belongs to QX-type G-19 of IBV, however, it was also closely associated with Duck-CoV and Pheasant-CoV. Abro et al. (60) carried bioinformatics of CoVs and found inter-species transmission of avian and mammalian CoVs that suggested inter-species transmission and were in agreement with our findings.

## Conclusion

Based on the S glycoprotein, SARS-CoV-2, SARS-CoV, Bats-CoV, and MERS-CoV have close genetic relationship. IBV can potentially infect wider range of bird species beyond their natural hosts chicken e.g., ducks, teal, partridge, turkeys, and pheasants. Duck-CoV, and Pheasant-CoV grouped together with IBV strain HH06 and other QX-type viruses, hence there is an imperative need to work further on the transmission mechanism of these CoVs. Purifying selection contributed predominately to the evolutionary process in selection pressure analysis. Sufficient chances of recombination and evolution of CoVs result in the rise of novel CoVs, which have great ability of intra-species transmission and zoonosis. Thus, their continuous emerging worldwide needs to be controlled more efficiently.

## Data Availability Statement

The datasets presented in this study can be found in online repositories. The names of the repository/repositories and accession number(s) can be found in the article/Supplementary Material.

## Ethics Statement

Ethical review and approval was not required for the study on human participants in accordance with the local legislation and institutional requirements. Written informed consent for participation was not required for this study in accordance with the national legislation and the institutional requirements.

## Author Contributions

GA and YZ: conceptualization. GA and GL: data curation. GA and XS: formal analysis. GL: funding acquisition, project administration, resources, and supervision. HC and XH: investigation. GA: methodology and writing–original draft. GA and MA: software. YR and XW: validation. GA, HC, and GL: writing–review and editing. All authors agreed to the final version.

## Funding

The National Natural Science Foundation of China under Grant (31172295 and 31272569) supported this research.

## Conflict of Interest

The authors declare that the research was conducted in the absence of any commercial or financial relationships that could be construed as a potential conflict of interest.

## Publisher's Note

All claims expressed in this article are solely those of the authors and do not necessarily represent those of their affiliated organizations, or those of the publisher, the editors and the reviewers. Any product that may be evaluated in this article, or claim that may be made by its manufacturer, is not guaranteed or endorsed by the publisher.

## Supplementary Material

Supplementary Table S1 The information of Coronaviruses protein and gene sequences used in bioinformatics.

Supplementary Table S2 Analysis and description of motif present in spike glycoprotein sequences of coronaviruses.

Supplementary Table S3 Evolutionary fingerprinting analysis of Coronaviruses proteins.

Supplementary Table S4 Maximum likelihood analysis of S protein for codon-by-codon positive selection.

Supplementary Figure S1 Dwarfism and hemorrhage of embryos 72 h post-infection with IBV HH06 at the left, the control embryo at the right.

Supplementary Figure S2 Confirmation of IBV Strain HH06 using IBV-N specific primers.
