# Supplementary material for: Molecular Characterization of Infectious Bronchitis Virus Strain HH06 Isolated in a Poultry Farm in Northeastern China
Source: Front Vet Sci. 2021 Dec 16;8:794228. doi: 10.3389/fvets.2021.794228 (PMC8716591; doi:10.3389/fvets.2021.794228)
Supplement: Supplementary Table S1 — The information of Coronaviruses protein and gene sequences used in bioinformatics. [file Table_1.DOCX]

| **Supplementary Table 1.** The information of Coronaviruses protein and gene sequences used in bioinformatics | | | | | | | | | | |
| --- | --- | --- | --- | --- | --- | --- | --- | --- | --- | --- |
| **Access. No** | **Strains/**  **Isolate** | **Organism** | **Genus** | **a.a** | **Host** | **CDS ID** | **Start** | **End** | **Country** |  |
|  |  |  | *Gamacorona viruses* |  |  |  |  | |  |  |
| QAT77248.1 | HH06* | Infectious Bronchitis Virus (IBV) | Our Strain | 1156 | *Gallus gallus* | MH181793.1 | 20367 | 23837 | China |  |
| QKV27964.1 | QX | IBV | QX-Type /G1-19 | 1165 | *Gallus gallus* | MN548289.1 | 20333 | 23830 | United Kingdom |  |
| QAA92387.1 | IBS130/2015 | IBV | QX-Type /G1-19 | 1165 | *Gallus gallus* | MG738155.1 | 20362 | 23859 | Malaysia |  |
| AYC44182.1 | CK/CH/HD/171018 | IBV | QX-Type /G1-19 | 1165 | *Gallus gallus* | MH020185.1 | 20371 | 23868 | China |  |
| ARS22420.1 | QIA-KR/D79/05 | IBV | QX-Type /G1-19 | 1165 | *Gallus gallus* | KU900740.1 | 20374 | 23871 | South Korea |  |
| ABI26423.1 | M41* | IBV | Mass- Type/G1-1 | 1162 | *Gallus gallus* | DQ834384.1 | 20375 | 23863 | USA |  |
| ACJ12834.1 | H52 | IBV | Mass -Type/G1-1 | 1162 | *Gallus gallus* | EU817497.1 | 20314 | 23802 | China |  |
| ACO37566.1 | H120 | IBV | Mass- Type/G1-1 | 1162 | *Gallus gallus* | FJ807652.1 | 20314 | 23802 | China |  |
| QIV13699.1 | TW2296/95vac | IBV | TW -Type/G1-7 | 1163 | *Gallus gallus* | MN128086.1 | 20421 | 23912 | Taiwan |  |
| ABG36787.1 | TW2575/98 | IBV | TW -Type/G1-7 | 1165 | *Gallus gallus* | DQ646405.2 | 20438 | 23935 | Taiwan |  |
| QJE49266.1 | ck/CH/LJX/2017/07 | IBV | TW -Type/G1-7 | 1165 | *Gallus gallus* | MN307884.1 | 20368 | 23865 | China |  |
| AVI69485.1 | ck/CH/LSC/99I | IBV | LSC 991-Type/G1-22 | 1167 | *Gallus gallus* | KY799582.1 | 20368 | 23871 | China |  |
| AEJ80238.2 | YN | IBV | LSC 991-Type/G1-22 | 1167 | *Gallus gallus* | JF893452.2 | 20361 | 23864 | China |  |
| ADI54955.1 | CQ04-1 | IBV | LSC 991-Type/G1-22 | 1167 | *Gallus gallus* | HM245924.1 | 20371 | 23874 | China |  |
| ACH72804.1 | SC021202 | IBV | LSC 991-Type/G1-22 | 1158 | *Gallus gallus*/ | EU714029.1 | 20361 | 23837 | China |  |
| AMH39305.1 | LDT3-A | IBV | LDT3A-TYPE/G1-18 | 1165 | *Anas crecca* | KR608272.1 | 20381 | 23878 | China |  |
| AAT70772.1 | partridge/GD/S14/2003* | IBV | LDT3A-TYPE/G1-18 | 1165 | *Perdix perdix Gallus gallus* | AY646283.1 | 20200 | 23697 | China |  |
| QOS02275.1 | IA1162/2020 | IBV | DMV-1639/G1-17 | 1166 | *Gallus gallus* | MW024789.1 | 20367 | 23867 | USA |  |
| QGM12378.1 | IBV/Ck/Can/17-035614 | IBV | DMV-1639/G1-17 | 1166 | *Gallus gallus* | MN512434.1 | 20403 | 23903 | Canada |  |
| QCX19619.1 | GA9977/2019 | IBV | DMV-1639/G1-17 | 1166 | *Gallus gallus* | MK878536.1 | 20371 | 23871 | USA |  |
| AYA44691.1 | ArkGA_P1 | IBV | Arkansas/G1-27 | 1168 | *Gallus gallus* | MH779856.1 | 20332 | 23838 | USA |  |
| AYA44701.1 | ArkGA_P20 | IBV | Arkansas/G1-27 | 1168 | *Gallus gallus* | MH779857.1 | 20332 | 23838 | USA |  |
| AGY56140.1 | 4/91 vaccine | IBV | 4/91-Type/G1-13 | 1164 | *Gallus gallus* | KF377577.1 | 20314 | 23808 | China |  |
| AJT47842.1 | ck/CH/LHB/130927 | IBV | 4/91-Type/G1-13 | 1164 | *Gallus gallus* | KP118880.1 | 20314 | 23808 | China |  |
| AJP16721.1 | ck/CH/LHB/121010 | IBV | 4/91-Type/G1-13 | 1164 | *Gallus gallus* | KP036503.1 | 20314 | 23808 | China |  |
| AAZ09202.1 | CK/CH/LDL97I/97 | IBV | LDL971-Type/G1-16 | 1166 | *Gallus gallus* | DQ068701.1 | complete | cds | China |  |
| ACJ50191.1 | 2992/02 | IBV | LDL971-Type/G1-16 | 1166 | *Gallus gallus* | EU822340.1 | begin | 3501 | Taiwan |  |
| AKF17724.1 | DK/GD/27/2014 | Duck coronavirus (DCoV) |  | 1191 | *Anas platyrhynchos* | KM454473.1 | 20318 | 23893 | China |  |
| AEO86768.1 | DK/CH/HN/ZZ2004* | Duck coronavirus (DCoV) |  | 1165 | **Anas platyrhynchos* | JF705860.1 | 20370 | 23867 | China |  |
| ALQ43515.1 | 080385d* | Turkey coronavirus (TCoV) |  | 1198 | *Meleagris gallopavo* | KR822424.1 | 20365 | 23961 | France |  |
| CBA13338.1 | FR080147c | Turkey coronavirus (TCoV) |  | 1198 | *Meleagris gallopavo* | FN434203.1 |  |  | France |  |
| AIL92592.1 | IN/421/10 | Turkey coronavirus (TCoV) |  | 1208 | *Meleagris gallopavo* | KF652218.1 |  |  | USA |  |
| ABW75138.1 | TCoV-ATCC | Turkey coronavirus (TCoV) |  | 1203 | embryonated turkey eggs (21 day)" | EU022526.1 | 20392 | 24003 | USA: Indiana |  |
| YP_001941166.1 | MG10 | Turkey coronavirus (TCoV) |  | 1226 | *Meleagris gallopavo* | NC_010800.1 | 20360 | 24040 | Canada |  |
| AIL92611.1 | MN/310/96 | Turkey coronavirus (TCoV) |  | 1203 | *Meleagris gallopavo* | KF652237.1 | complete cds |  | USA |  |
| AIL92612.1 | NC/1440/99 | Turkey coronavirus (TCoV) |  | 1207 | *Meleagris gallopavo* | KF652238.1 | complete cds |  | USA |  |
| AIL92595.1 | IN/834/04 | Turkey coronavirus (TCoV) |  | 1209 | *Meleagris gallopavo* | KF652221.1 | complete cds |  | USA |  |
| AAU09490.1 | LKQ3* | peafowl corona virus (PeFCoV) |  | 1162 | *Pavo cristatus* | AY702085.1 | begin | 3489 | China |  |
| AAZ85066.1 | PSH050513* | Pigeon coronavirus (PiCoV) |  | 1167 | *Columba livia* | DQ160004.1 | 67 | 3570 | China |  |
| QCB65097.1 | Cambridge Bay* | Canada goose coronavirus (GCoV) |  | 1184 | *Branta canadensis* | MK359255.1 | 20069 | 23623 | Canada |  |
| QDY92359.1 | MW18 | Infectious Bronchitis Virus (IBV) |  | 1180 | *Tadorna tadornoides* | MK204411.1 | 20472 | 24014 | Australia |  |
| QDA76255.1 | ph/China/I0710* | Pheasant coronavirus (PhCoV) |  | 1165 | *Phasianus colchicus* | MK423876.1 | 20372 | 23869 | China |  |
| AAU14248.1 | LDT3* | Infectious Bronchitis Virus (IBV) |  | 1165 | *Anas crecca* | AY702975.1 | began | 3498 | China |  |
| ABW87820.1 | SW1* | Beluga whale coronavirus SW1 |  | 1472 | *Delphinapterus leucas* *Delphinapterus leucas* | EU111742.1 | 20450 | 24868 |  |  |
| QII89019.1 | 37112-1* | Bottlenose dolphin coronavirus |  | 1482 | *Tursiops truncatus* | MN690608.1 | 20450 | 24898 | USA |  |
| AHB63481.1 | CF090325* | Bottlenose dolphin coronavirus HKU22 |  | 1493 |  | KF793824.1 | 20446 | 24927 |  |  |
| AFH55111.1 | DF-2 R3i | Feline coronavirus (FCoV) | *Alphacorona viruse* | 1454 | *Felis catus* | JQ408980.1 | 20436 | 24800 |  |  |
| AAY32596.1 | FIPV 79-1146 | Feline coronavirus (FCoV) |  | 1452 | *Felis catus* | DQ010921.1 | 20206 | 24564 | USA |  |
| QRN75109.1 | 79-1146_CA | Feline coronavirus (FCoV) |  | 1452 | *Felis catus* | MW030109.1 | 20437 | 24795 | USA |  |
| AFG19742.1 | TN-449* | Canine coronavirus (CCoV) |  | 1454 | *Canis familiaris* | JQ404410.1 | 20367 | 24731 |  |  |
| ASB15738.1 | HLJ-071 | Canine coronavirus (CCoV) |  | 1454 | *Canis familiaris* | KY063616.1 | 20284 | 24648 | China |  |
| QJI07172.1 | B858_ZJ_2019 | Canine coronavirus (CCoV) |  | 1453 | *Canis familiaris* | MT114547.1 | complete cds |  | China |  |
| CAA42686.1 | PRCV* 86/137004 | Porcine respiratory coronavirus (PRCV) |  | 1225 | *Sus domesticus* | X60089.1 | 394 | 4071 | UK |  |
| AKV62755.1 | OH7269 | Porcine respiratory coronavirus (PRCV) |  | 1232 | *Sus domesticus* | KR270796.1 | 20353 | 24051 | USA |  |
| ASV64295.1 | TGEV-138* | Transmissible gastroenteritis virus (TGEV) |  | 1449 | *Sus domesticus* | KX900395.1 | 20353 | 24702 | USA |  |
| AAT00645.1 | HN2002 | Transmissible gastroenteritis virus (TGEV) |  | 1449 | *Sus domesticus* | AY587882.1 |  |  | China |  |
| ABI30278.1 | DR13* | Porcine epidemic diarrhea virus (PEDV) |  | 1383 | *Sus domesticus* | DQ862099.1 | 11 | end | South Korea |  |
| ABM64776.1 | LZC | Porcine epidemic diarrhea virus (PEDV) |  | 1383 | *Sus domesticus* | EF185992.1 | 20638 | 24789 | China |  |
| AXX83351.1 | YC-55* | Human coronavirus OC43 (HCoV OC43) | *Betacorona Viruse A* | 1362 | *Homo sapiens* | MG197718.1 | 23544 | 27640 | China |  |
| QRK03812.1 | OC43/China | Human coronavirus OC43 (HCoV OC43) |  | 1356 | *Homo sapiens* | MW532108.1 | 23615 | 27685 | China |  |
| ANZ78841.1 | 4400A/2015 | Human coronavirus OC43 (HCoV OC43) |  | 1362 | *Homo sapiens* | KU745540.1 |  |  | China |  |
| ABP87990.1 | NC99* | Equine coronavirus (ECoV) |  | 1363 | Lab host-HRT-18G cells | EF446615.1 | 23744 | 27835 |  |  |
| BAJ52885.1 | Tokachi09 | Equine coronavirus (ECoV) |  | 1363 | *Equus caballus* adult | AB555560.1 | begin | 4092 | Japan |  |
| BAS18856.1 | Obihiro12-1 | Equine coronavirus (ECoV) |  | 1363 | *Equus caballus* adult | LC061273.1 | 23708 | 27799 | Japan |  |
| BBM60917.1 | GIF-1* | Bovine coronavirus (BCoV) |  | 1363 |  | LC494126.1 | 23614 | 27705 | Japan |  |
| AGO98871.1 | SWE/N/05-1 | Bovine coronavirus (BCoV) |  | 1363 | *Bubalus bubalis* calf | KF169920.1 | complete cds |  | Sweden |  |
| ADP21336.1 | Himalayan Tahr1 | Bovine coronavirus (BCoV) |  | 1363 | *Hemitragus jemlahicus* | HM573327.1 | complete cds |  | South Korea |  |
| AAP72986.1 | HSR 1 | SARS coronavirus (SARS-CoV) | *Betacorona Viruse B* | 1255 | *Homo sapiens* | AY323977.2 | 21492 | 25259 | Italy |  |
| AAR07630.1 | BJ302 | SARS coronavirus (SARS-CoV) |  | 1255 | *Homo sapiens* | AY429078.1 |  |  | China |  |
| AAU81608.1 | SARS-CoV* | SARS coronavirus (SARS-CoV) |  | 1255 | *Homo sapiens* | AY714217.1 | 21492 | 25259 | USA |  |
| AAP33697.1 | Frankfurt 1 | SARS coronavirus (SARS-CoV) |  | 1255 | *Homo sapiens* | AY291315.1 | 21492 | 25259 | Germany |  |
| AAR23250.1 | Sino1-11 | SARS coronavirus (SARS-CoV) |  | 1255 | *Homo sapiens* | AY485277.1 | 21492 | 25259 | China |  |
| YP_009825051.1 | Tor2 | SARS coronavirus (SARS-CoV) |  | 1255 | *Homo sapiens* | NC_004718.3 | 21492 | 25259 | Canada |  |
| YP_009724390.1 | Wuhan-Hu-1* | SARS-COV2 |  | 1273 | *Homo sapiens* | NC_045512.2 | 21563 | 25384 | China |  |
| BCN86353.1 | JP_Hiro66017 | SARS-COV2 |  | 1282 | *Homo sapiens* | LC594644.1 | 21532 | 25380 | Japan |  |
| QJF75467.1 | MI-MDHHS-SC20058 | SARS-COV2 |  | 1273 | *Homo sapiens* | MT412183.1 | 21525 | 25346 | USA |  |
| QJS39567.1 | mink/NED/NB02_07KS | SARS-CoV-2 |  | 1273 | *Mustela lutreola* | MT457396.1 | 21550 | 25371 | Netherlands |  |
| QJR85953.1 | AUS/VIC82 | SARS-CoV-2 |  | 1273 | *Homo sapiens* | MT450992.1 | 21524 | 25345 | Australia |  |
| QIZ16509.1 | TUR/ERAGEM-001 | SARS-CoV-2 |  | 1273 | *Homo sapiens* | MT327745.1 | 21559 | 25380 | Turkey |  |
| QPF48560.1 | POL/PL_MCB_43 | SARS-CoV-2 |  | 1273 | *Homo sapiens* | MW273825.1 | 21538 | 25359 | Poland |  |
| QIA98583.1 | IND/166 | SARS-CoV-2 |  | 1273 | *Homo sapiens* | MT050493.1 | 21543 | 25364 | India |  |
| QIU81873.2 | Beijing_IME-BJ05 | SARS-CoV-2 |  | 1273 | *Homo sapiens* | MT291835.2 | 21538 | 25359 | China |  |
| QQK89979.1 | IRN/K1r-145 | SARS-CoV-2 |  | 1273 | *Homo sapiens* | MW440440.1 | complete cds |  | Iran |  |
| YP_009047204.1 | HCoV-EMC | MERS-CoV | *Betacorona Viruse C* | 1353 | *Homo sapiens* | NC_019843.3 | 21456 | 25517 | Saudi Arabia |  |
| AKN11072.1 | MERS-CoV/KOR/KNIH* | MERS-CoV |  | 1353 | *Homo sapiens* | KT182954.1 |  |  | South Korea |  |
| AHI48572.1 | Riyadh_14 | MERS-CoV |  | 1353 | *Homo sapiens* | KJ156934.1 | 21405 | 25466 | Saudi Arabia |  |
| QGW51521.1 | Al-Hasa-SA4047 | MERS-CoV |  | 1353 | *Camelus dromedarius* | MN654983.1 | 21456 | 25517 | Saudi Arabia |  |
| QHB14973.1 | KFU-HKU-R | MERS-CoV |  | 1353 | *Camelus dromedarius* | MN758608.1 | 21456 | 25517 | Saudi Arabia |  |
| ASU90340.1 | UAE_B40 | MERS-CoV |  | 1353 | *Camelus dromedarius* | MF598632.1 | 21456 | 25517 | UAE |  |
| QSQ01650.1 | PrC31* | Bat coronavirus | *Betacorona Viruse D* | 1246 | *Rhinolophus blythi* | MW703458.1 | 21521 | 25261 | China |  |
| QQM18864.1 | RacCS203 | Bat coronavirus |  | 1227 | *Rhinolophus acuminatus* | MW251308.1 | 21562 | 25245 | Thailand |  |
| Q3LZX1.1 | HKU3* | Bat coronavirus |  | 1242 | *Rhinolophus sinicus* |  |  |  | China |  |
| ARO76382.1 | F46 | SARS-related Cov |  | 1236 | Chiroptera | KU973692.1 | 21492 | 25202 | China |  |
| ANA96027.1 | JTMC15 | Bat coronavirus |  | 1236 | *Rhinolophus ferrumequinum* | KU182964.1 | 20894 | 24604 | China |  |
| ASO66810.1 | 16BO133 | Bat coronavirus |  | 1236 | *Rhinolophus ferrumequinum* | KY938558.1 | 20901 | 24611 | South Korea |  |
| YP_002308506.1 | HKU13-3514* | Munia coronavirus | *Deltacorona virus* | 1156 | *Lonchura striata* | NC_011550.1 | 19499 | 22969 | China |  |
| AWV67125.1 | ISU42824* | Sparrow deltacoronavirus |  | 1157 | *Passer domesticus* | MG812377.1 | 19260 | 22733 | USA |  |
| AWV67107.1 | ISU690-4 | Sparrow deltacoronavirus |  | 1156 | *Passer domesticus* | MG812375.1 | 19279 | 22749 | USA |  |
| BBC54852.1 | 411F* | Quail coronavirus UAE-HKU30 |  | 1158 | *Coturnix coturnix* | LC364345.1 | 19294 | 22770 | UAE |  |
| BBC54861.1 | 1101F | Quail coronavirus UAE-HKU30 |  | 1158 | *Coturnix coturnix* | LC364346.1 | 19293 | 22769 | UAE |  |
| ACN89723.1 | SA59/RJHM* | Murine coronavirus |  | 1324 | *Mus musculus* | FJ647222.1 | 23896 | 27870 | USA |  |
| AGT17749.1 | icA59/ns2M | Murine coronavirus |  | 1324 | *Mus musculus* | KF268339.1 | 23904 | 27878 | USA |  |
| QSJ02954.1 | MHV-3/UNICAMP | Murine coronavirus |  | 1383 | *Mus musculus* | MW620427.1 | 23730 | 27881 | Brazil |  |
| ACN89743.1 | MHV-3 | Murine coronavirus |  | 1377 | *Mus musculus* | FJ647224.1 | 23883 | 28016 | USA |  |
| ACN89705.1 | RA59/SJHM | Murine coronavirus |  | 1376 | *Mus musculus* | FJ647220.1 | 23890 | 28020 |  |  |
| ATP66750.1 | RtAp-CoV/Tibet2014 | Rodent coronavirus |  | 1358 | *Apodemus peninsulae* | KY370047.1 | 23818 | 27894 | China |  |
| YP_009755834.1 | RtMruf-CoV-2* | Rodent coronavirus |  | 1361 | *Myodes rufocanus* | NC_046954.1 | 23757 | 27842 | China |  |
| ATP66733.1 | RtAs-CoV/IM2014 | Rodent coronavirus |  | 1370 | *Allactaga sibirica* | KY370044.1 | 24757 | 28869 | China |  |
| QCI62295.1 | CH-HA2-2017* | Porcine deltacorona virus |  | 1159 | *Sus domesticus* | MK040454.1 |  |  | China |  |
| AXP32216.1 | HNZK-02 | Porcine deltacorona virus |  | 1159 | *Sus domesticus* | MH708123.1 | 19324 | 22803 | China |  |

a.a=amino acid, CDS= coding sequences, *=strain used in multiple sequence alignment
