# Supplementary material for: Molecular Characterization of Infectious Bronchitis Virus Strain HH06 Isolated in a Poultry Farm in Northeastern China
Source: Front Vet Sci. 2021 Dec 16;8:794228. doi: 10.3389/fvets.2021.794228 (PMC8716591; doi:10.3389/fvets.2021.794228)
Supplement: Supplementary Table S2 — Analysis and description of motif present in spike glycoprotein sequences of coronaviruses. [file Table_2.DOCX]

**Table S 2.** Analysis and description of motif present in Spike glycoprotein sequences of coronaviruses

| **No** | **Sequence** | **Width, bp** | **PFAM Description** |
| --- | --- | --- | --- |
| 1 | LATQKINECVKSQSKRYGFCGNGRHVLSIPQNAPNGIVFIH | 41 | CoV S Glycoprotein |
| 2 | LNDSLIBLEELGKYETYIKWPWYVWLAIG | 29 | CoV S Glycoprotein |
| 3 | VNKQAQALNELMNQLSNNFGAISSSJQDIYSRLDALZADAQVDRLITGRL | 50 | CoV S Glycoprotein |
| 4 | GITAAAAIPFATQVQARINGLGITQSVLLENQKLIANSFNKAIGHIQEGF | 50 | CoV S Glycoprotein |
| 5 | IPTNFTJSVTTEYIQTRMDKVQIDCAQYVCGDSLECRKLLQQYGSFCDNI | 50 | CoV S Glycoprotein |
| 6 | GTLRDLICAQYYNGJLVLPPVJTADMIAMYTASLVGGMAFG | 41 | CoV S Glycoprotein |
| 7 | GWVFFMTGCCGCCCGCFGIIPLMSKCGKKSSYYTTFDNDVVTEQYRPKKS | 50 | CoV S Glycoprotein |
| 8 | GRGIFVQVNGSWYITARBMYMPRDITAGBIVVLTSCQVNYV | 41 | CoV S Glycoprotein |
| 9 | RSFIEDLLFNKVETVGLGTVD | 21 | CoV S Glycoprotein |
| 10 | HNYTNJTLNVCVBYNIYGRTGQGVITNVT | 29 | CoV S Glycoprotein |
