# Supplementary material for: Molecular Characterization of Infectious Bronchitis Virus Strain HH06 Isolated in a Poultry Farm in Northeastern China
Source: Front Vet Sci. 2021 Dec 16;8:794228. doi: 10.3389/fvets.2021.794228 (PMC8716591; doi:10.3389/fvets.2021.794228)
Supplement: Supplementary Table S3 — Evolutionary fingerprinting analysis of Coronaviruses proteins. [file Table_3.DOCX]

**Table S 3.** Evolutionary fingerprinting analysis of Coronaviruses proteins

| **Class** | **α** | | | | **β** | | | | **ω** | **Weight** | | | |
| --- | --- | --- | --- | --- | --- | --- | --- | --- | --- | --- | --- | --- | --- |
|  | **MLE** | **2.5%** | **Median** | **97.5%** | **MLE** | **2.5%** | **Median** | **97.5%** | **MLE** | **MLE** | **2.5%** | **Median** | **97.5%** |
| 1 | 0.75 | 0.68 | 0.75 | 0.81 | 0.003 | 0.00 | 0.00 | 0.00 | 0.004 | 0.060 | 0.05 | 0.06 | 0.07 |
| 2 | 1.52 | 1.32 | 1.53 | 1.76 | 0.034 | 0.03 | 0.03 | 0.04 | 0.022 | 0.031 | 0.02 | 0.03 | 0.04 |
| 3 | 0.88 | 0.81 | 0.88 | 0.96 | 0.104 | 0.09 | 0.10 | 0.11 | 0.117 | 0.179 | 0.15 | 0.18 | 0.22 |
| 4 | 1.04 | 0.97 | 1.04 | 1.12 | 0.286 | 0.27 | 0.29 | 0.30 | 0.275 | 0.226 | 0.19 | 0.23 | 0.27 |
| 5 | 0.59 | 0.54 | 0.59 | 0.65 | 0.046 | 0.04 | 0.05 | 0.05 | 0.077 | 0.088 | 0.07 | 0.09 | 0.11 |
| 6 | 1.21 | 1.11 | 1.20 | 1.30 | 0.182 | 0.17 | 0.18 | 0.20 | 0.151 | 0.187 | 0.15 | 0.19 | 0.23 |
| 7 | 0.61 | 0.55 | 0.61 | 0.66 | 0.159 | 0.14 | 0.16 | 0.17 | 0.262 | 0.131 | 0.10 | 0.13 | 0.17 |
| 8 | 1.52 | 1.33 | 1.52 | 1.72 | 0.475 | 0.42 | 0.48 | 0.53 | 0.312 | 0.079 | 0.06 | 0.08 | 0.11 |
| 9 | 2.01 | 1.34 | 1.99 | 2.91 | 1.091 | 0.74 | 1.08 | 1.59 | 0.543 | 0.018 | 0.01 | 0.02 | 0.03 |

E[α] = 1, E[β] = 0.200418, E[ω] = 0.187761, Cov (α,β) = 0.702138
