# Supplementary material for: Molecular Characterization of Infectious Bronchitis Virus Strain HH06 Isolated in a Poultry Farm in Northeastern China
Source: Front Vet Sci. 2021 Dec 16;8:794228. doi: 10.3389/fvets.2021.794228 (PMC8716591; doi:10.3389/fvets.2021.794228)
Supplement: Supplementary Table S4 — Maximum likelihood analysis of S protein for codon-by-codon positive selection. [file Table_4.DOCX]

| **Codon No.** | **Triplet** | **Syn (s)** | **Nonsyn (n)** | **Syn sites (S)** | **Non-syn sites (N)** | **dS** | **dN** | **dN-dS** | **P-value** |
| --- | --- | --- | --- | --- | --- | --- | --- | --- | --- |
| 26 | AGT | 12.0 | 32.00 | 0.87 | 2.08 | 13.84 | 15.39 | 1.55 | 0.45 |
| 56 | CAT | 9.00 | 36.00 | 0.70 | 2.24 | 12.77 | 16.04 | 3.27 | 0.34 |
| 68 | TTA | 9.50 | 23.50 | 0.93 | 2.02 | 10.24 | 11.66 | 1.42 | 0.45 |
| 132 | GAT | 6.67 | 26.33 | 0.64 | 2.24 | 10.38 | 11.76 | 1.38 | 0.48 |
| 151 | AAT | 6.00 | 23.00 | 0.71 | 2.24 | 8.51 | 10.26 | 1.75 | 0.44 |
| 153 | TCA | 8.00 | 28.00 | 0.86 | 2.06 | 9.35 | 13.60 | 4.24 | 0.23 |
| 167 | GAC | 10.00 | 32.00 | 0.75 | 2.15 | 13.31 | 14.85 | 1.55 | 0.46 |
| 168 | AAG | 7.00 | 25.00 | 0.69 | 2.19 | 10.21 | 11.42 | 1.20 | 0.49 |
| 173 | AGT | 8.50 | 29.50 | 0.70 | 2.19 | 12.09 | 13.47 | 1.38 | 0.48 |
| 225 | ATT | 8.20 | 19.83 | 0.98 | 2.02 | 8.35 | 9.84 | 1.49 | 0.43 |
| 233 | TTT | 5.25 | 26.75 | 0.69 | 2.22 | 7.61 | 12.06 | 4.46 | 0.23 |
| 360 | AAT | 5.50 | 15.50 | 0.90 | 2.10 | 6.12 | 7.38 | 1.27 | 0.46 |
| 662 | AAT | 5.50 | 21.50 | 0.65 | 2.17 | 8.49 | 9.89 | 1.40 | 0.48 |
| 708 | CAG | 6.75 | 26.25 | 0.75 | 2.18 | 9.02 | 12.02 | 3.00 | 0.33 |
| 718 | GTT | 2.50 | 17.50 | 0.77 | 2.19 | 3.26 | 7.98 | 4.73 | 0.14 |
| 794 | GGT | 4.00 | 26.00 | 0.73 | 2.19 | 5.46 | 11.89 | 6.43 | 0.10 |
| 866 | AAG | 3.50 | 14.50 | 0.65 | 2.14 | 5.39 | 6.78 | 1.39 | 0.48 |

**Table S4.** Maximum likelihood analysis of S protein for codon-by-codon positive selection
