# Supplementary figures and images for: Molecular Characterization of Infectious Bronchitis Virus Strain HH06 Isolated in a Poultry Farm in Northeastern China
Source: Front Vet Sci. 2021 Dec 16;8:794228. doi: 10.3389/fvets.2021.794228 (PMC8716591; doi:10.3389/fvets.2021.794228)

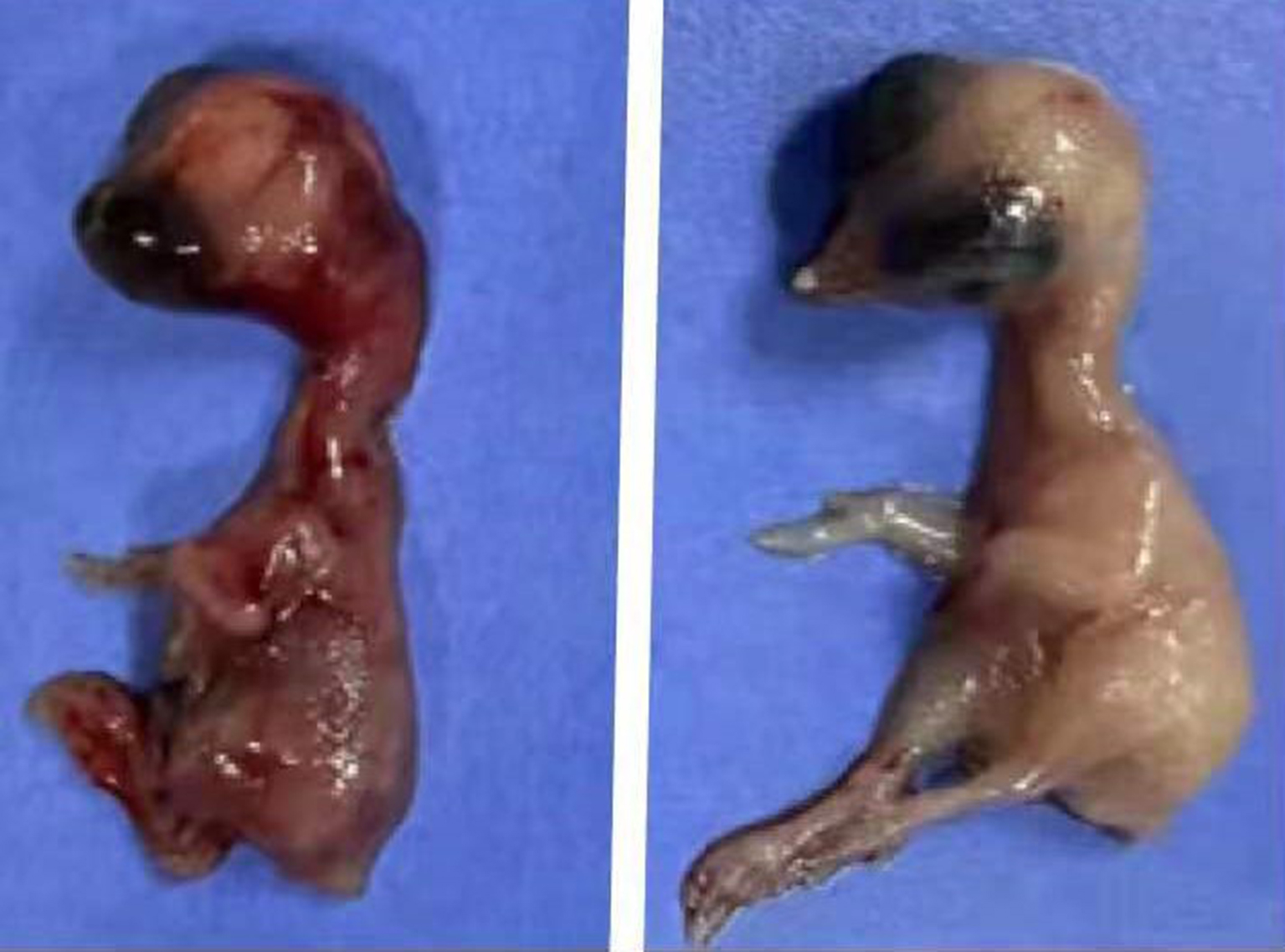

Supplement: Supplementary Figure S1 — Dwarfism and hemorrhage of embryos 72 h post-infection with IBV HH06 at the left, the control embryo at the right. [file Image_1.JPEG]

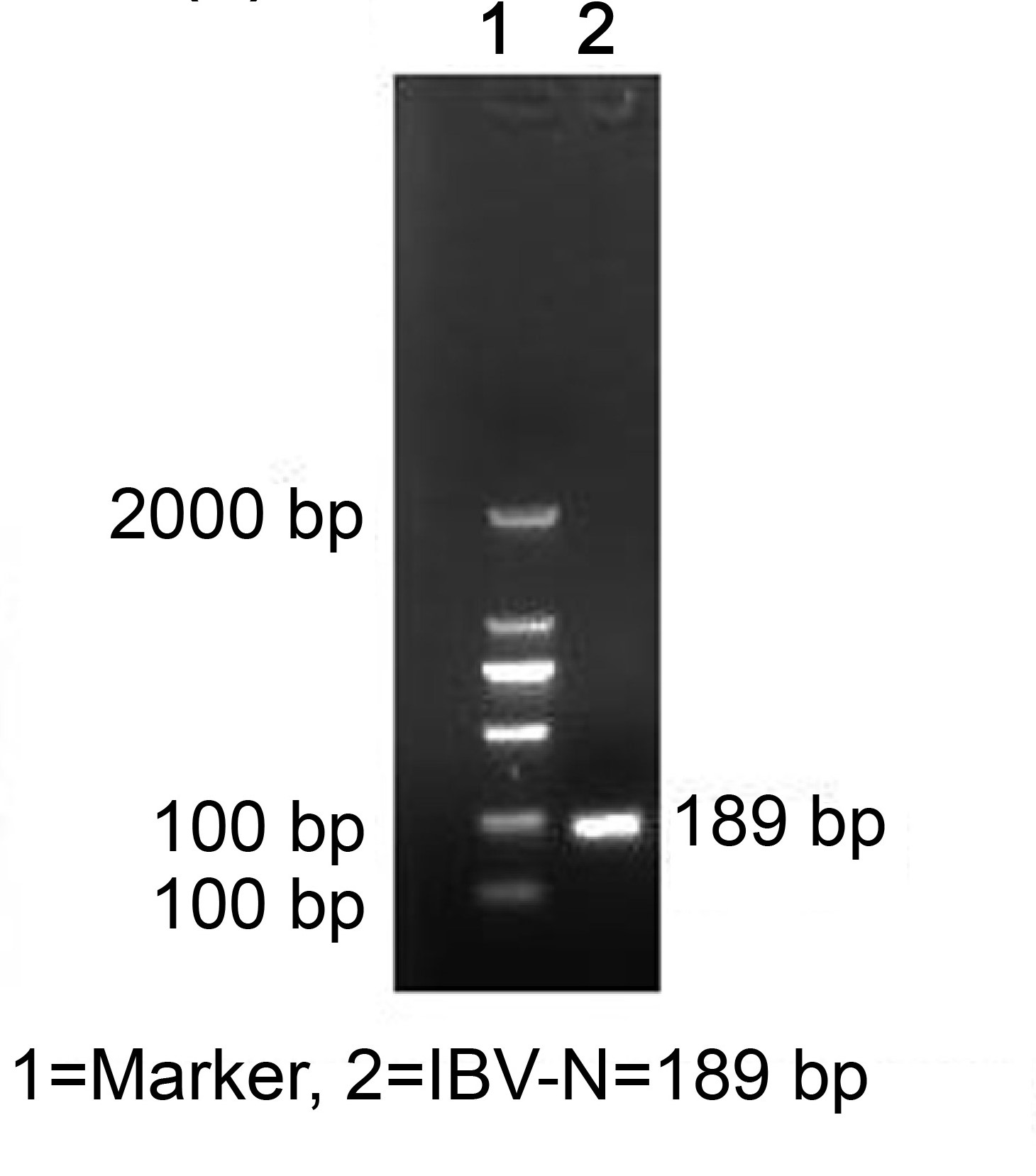

Supplement: Supplementary Figure S2 — Confirmation of IBV Strain HH06 using IBV-N specific primers. [file Image_2.JPEG]
